# Impact of carbon sources in airport de-icing compounds on the growth of *Sphaerotilus natans*

**DOI:** 10.3389/fmicb.2024.1320487

**Published:** 2024-10-23

**Authors:** Benjamin Exton, Francis Hassard, Angel Medina-Vaya, Robert C. Grabowski

**Affiliations:** ^1^Faculty of Engineering and Applied Sciences, Cranfield University, Cranfield, United Kingdom; ^2^Institute for Nanotechnology and Water Sustainability, University of South Africa, Johannesburg, South Africa

**Keywords:** undesirable river biofilm, airport de-icer, *Sphaerotilus natans*, microcosm, growth kinetics, sewage fungus

## Abstract

Airport de-icing has been linked with the growth of undesirable river biofilms (URBs, formerly “sewage fungus”), a manifestation of organic pollution causing long-term ecological damage to watercourses. URBs are a polymicrobial community, with one key taxon standing out in literature: *Sphaerotilus natans*, a filamentous bacterium also found in sewage treatment and activated sludges. An industry often implicated in causing URBs is airport de-icing, with large biofilms often developing downstream of airport discharges in winter months. However, it is not yet clear which de-icers may cause URBs and how they influence growth. Therefore, specific objectives were to (i) determine which freeze-point depressants (FPDs) can be utilized by *S. natans*; (ii) examine differences in the growth kinetics between FPDs; and (iii) compare pure-FPDs to commercial airport de-icers (CADs) as carbon sources, to determine impacts of additives. This study employed a turbidimetric micro-batch culture design to conduct microbial growth experiments, using *S. natans* and a minimal medium supplemented with airport de-icer as the carbon source. Equimolar carbon concentrations were used to compare the effects of common FPDs and CADs – each containing a specific FPD. Growth was assessed via optical density (OD_600_) measurements, from which time-to-detection, maximum rate of change, and maximum optical density were derived and kinetics inferred. *S. natans* was found to grow effectively on all FPDs tested, although the microbial yield was heavily dependent on the carbon concentration for all FPDs and CADs. Sodium acetate generated the quickest growth, with the lowest TTD (lag-time) for all but the lowest concentrations tested. Propylene glycol produced the greatest maxOD (total growth), whereas ethylene glycol had a higher limiting concentration for maxROC (growth rate). The mixture of compounds and additives in commercial products did not significantly impact the growth of *S. natans*. This research provides evidence from controlled laboratory experiments that airport de-icers support the growth of *S. natans*. The differences in growth kinetics observed for the FPDs and CADs could inform improved mitigation or treatment to reduce the incidence and ecological impacts of URBs.

## Graphical abstract

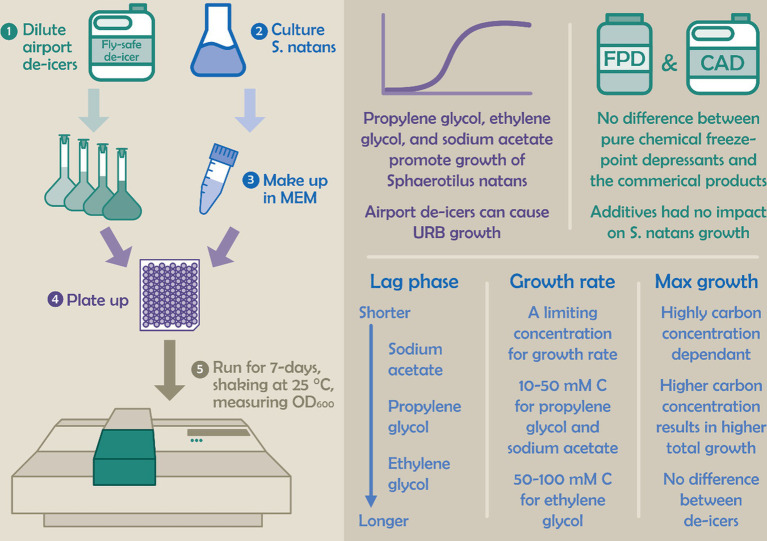

## Highlights

- The first definitive link between airport de-icers and URB growth
- *S. natans* grew using propylene glycol, ethylene glycol and sodium acetate freeze-point depressants
- Sodium acetate was consumed earlier and faster, followed by propylene glycol, then ethylene glycol
- Greater maxOD with propylene glycol and higher rate limiting concentration with ethylene glycol
- There were minimal differences between commercial products and pure chemicals indicating additives did not hinder microbial growth.

## 1 Introduction

Undesirable river biofilms (URBs), also referred to as “sewage fungus,” are a consequence of organic pollution in fluvial systems (Harrison and Heukelekian, 1958; Curtis and Harrington, 1971; Gray, 1985; Quinn and Mcfarlane, 1985). These biofilms are widely distributed, especially in countries lacking established wastewater treatment infrastructure, and cause significant ecological impact on affected watercourses (Hirsch, 1958; Hynes, 1960; Smith and Kramer, 1963; Curtis, 1969; Curtis and Curds, 1971; Gray, 1987; Lemly, 1998). URBs serve as a visual bioindicator of poor water quality (Quinn and Mcfarlane, 1985) and can provide valuable data regarding overall river health in the face of diffuse and intermittent organic pollution (Villeneuve et al., 2011; Battin et al., 2016; Sanhita, 2019), for example, spills due to combined sewer overflows. Whilst URBs proliferate in rivers with poor water quality, they also contribute to further degradation in river health. For instance, they consume dissolved oxygen (DO) and nutrients, overgrow riverbeds and lead to significant loss of aquatic fauna and flora (Gray, 1985; Pillard, 1995), including macroinvertebrates (Turnbull and Bevan, 1995), fish species (Curtis, 1969; United States Environmental Protection Agency, 2000) and natural plant life (Hartwell et al., 1995). Moreover, the growth of URBs often results in complaints and disengagement from river users and stakeholders due to their unappealing appearance, olfactory issues, and the subsequent loss of recreational and ecosystem services (Koryak et al., 1998; ACRP, 2008). URBs are assumed to be an older problem, and this is reflected in the limited scientific research since the 1980’s, however, this is not the case (Exton et al., 2024). In the UK, there is substantial evidence to suggest that URBs remain highly prevalent (Irish Examiner, 2016; AirportWatch, 2019; BBC News, 2019b, 2019a, 2020, 2021; Somerset Live, 2019; ITV News, 2021; Oxford Mail, 2021; Fish Legal, 2022; GB News, 2022; Leicestershire Live, 2022; Nottinghamshire Live, 2022; The Guardian, 2022, 2023; Yahoo Sports, 2023). Recent interrogation of reporting data by the environmental regulator in England (Environment Agency) revealed 6,020 incidents of URBs were recorded in England from 2000 to 2020 and that this was known to be an underrepresentation of the true number, evidenced by the above media reports that were not included in Environment Agency data (Exton et al., 2024). It is also clear that a wide variety of industries and land use changes influence the frequency of URB proliferation (Exton et al., 2024). Whilst the specific causes of URBs are variable, catchment specific, and usually not directly attributable, one contaminant group implicated in URB growth is runoff from airport anti- and de-icer application. This association has been highlighted by local action groups (Citizen Crane, 2020), airport operators (Heathrow Airport Ltd, 2018; Ricardo, 2018), and local/national news (AirportWatch, 2019; BBC News, 2019a; Financial Times, 2022; Nottinghamshire Live, 2022), as well as in scientific literature (ACRP, 2014; Nott et al., 2020; Exton et al., 2023). Despite these suggested connections, a direct causal link between the URB growth and airport anti- and de-icers as a nutrient source has yet to be conclusively established or demonstrated empirically.

*Sphaerotilus natans*, a filamentous, rod-shaped, sheath-forming bacterium with sub-polar flagella in the family *Comamonadaceae* (Kämpfer and Spring, 2015) and is one of the key species frequently found in URBs (Butcher, 1932; Harrison and Heukelekian, 1958; Phaup, 1968; Curtis, 1969; Geatches et al., 2014). A significant amount of research into URBs was conducted between the 1950s and 1980s, often using *S. natans* as a representative taxon for the polymicrobial URB community (Stokes, 1954; Dondero, 1961; Mulder, 1964; Phaup and Gannon, 1967; Mueller and Litsky, 1968; Pellegrin et al., 1999). Recent molecular ecology analysis by Nott et al. (2020) applied microarray analysis and shotgun metagenomic sequencing, to URB outbreaks associated with airport de-icer (Nott et al., 2020). Whilst Exton et al. (2023) used 16S rRNA amplicon sequencing to discover that *Sphaerotilus* was either absent or very rare prior to airport de-icing activity (0.1% relative abundance) but became dominant (≥11.6%) in a subsequent URB growth (Exton et al., 2023). Other notable taxa include the genera *Zoogloea*, *Beggiatoa*, and *Rhodoferax* (Curtis, 1969; Geatches et al., 2014; Exton et al., 2023). However, despite these findings and the suspected links between airport de-icing activities and URB growth, no direct assessment of URB taxa growth using freeze-point depressants (FPDs) or commercial airport de-icer (CAD) formulations have been undertaken to date.

Substantial quantities of anti- and de-icers are used to ensure safe airport operation (Castro et al., 2005; United States Environmental Protection Agency, 2012; Freeman et al., 2015; Secretary of State for Transport, 2018; ACRP, 2020). However, heavy rainfall mobilizes the highly soluble substances causing them to enter waterways through runoff (Corsi et al., 2001; ACRP, 2020). This runoff travels through airport drainage networks and treatment systems before being discharged into receiving waters and the biosphere. Despite only constituting a small volume of receiving waters, airport anti- and de-icers have significant impacts on water quality (Turnbull and Bevan, 1995), including promoting the growth of ecosystem-dominating URBs. Although anti- and de-icers serve slightly different purposes, their chemical compositions are very similar. In this paper, the commercial products used by airports will be collectively referred to as CADs for the sake of brevity and euphony. The FPDs are the active ingredients in anti- and de-icers, creating a eutectic solution that lowers the freezing point of water (Fischel, 2001; Stefl et al., 2014; Schweigert and Poissonnier, 2016). Propylene glycol is often used in aircraft CADs, whilst CADs for ground application typically use ethylene glycol or formate/acetate salts as their FPDs (Ramakrishna and Viraraghavan, 2005; Samuels et al., 2006) (Table 1). Aircraft CADs are classified into different types based on their specific use cases. Type I CADs are exclusively for de-icing aircraft; Type II CADs are generally used for de-icing as well but provide more extended anti-icing properties than Type I; and Type IV CADs are primarily anti-icers but can be used for de-icing (International Standards Organization (ISO), 2007b; International Standards Organization (ISO), 2007a; International Standards Organization (ISO), 2020; Kilfrost LTD, 2019f; Kilfrost LTD, 2019e; Kilfrost LTD, 2019d; Kilfrost LTD, 2019c; Kilfrost LTD, 2019b; Kilfrost LTD, 2019a). Past research has suggested that *S. natans* can utilize low molecular weight alcohols, sugars, and acetate salts as carbon sources (Curtis et al., 1971), indicating that most FPD compounds should foster growth. However, formate salts reportedly cannot support growth when used as a carbon source in wastewater treatment processes including secondary treatment (i.e., the activated sludge process), and where filamentous bacterial growth has been extensively studied (Gulez et al., 2009).

**Table 1:** Overview of commonly used commercial airport de-icers (CADs), the freeze-point depressant (FPD) incorporated in each, their respective applications, and related chemical data.

	Commercial product	FPD	State	Use	Type	Chemical structure	M_W_ (g/mol)	log(KoW)
Aircraft	Kilfrost DF Plus (Kilfrost LTD, 2019f)	Propylene glycol	Liquid	Aircraft de-icing	Type I	CC(O)CO	76.095	−0.92 (Hansch et al., 1995)
	Kilfrost ABC-K Plus (Kilfrost LTD, 2019d)	Propylene glycol	Liquid	Primarily aircraft de-icing but can provide anti-icing protection	Type II	CC(O)CO	76.095	−0.92 (Hansch et al., 1995)
	Kilfrost ABC-S Plus (Kilfrost LTD, 2019e)	Propylene glycol	Liquid	Primarily aircraft anti-icing but can be used for de-icing	Type IV	CC(O)CO	76.095	−0.92 (Hansch et al., 1995)
Ground	Konsin	Ethylene glycol	Liquid	Runway de-icing	–	OCCO	62.068	−1.36 (Hansch et al., 1995)
	Clearway F1 (Kilfrost LTD, 2019b)	Potassium formate	Liquid	Runway, taxiway, and apron de-icing	–	C(=O)[O-].[K+]	84.115	–
	Clearway SF3 (Kilfrost LTD, 2019c)	Sodium formate	Solid	Snow and ice melt	–	[Na+].[O-]C=O	68.007	–
	Clearway 6S (Kilfrost LTD, 2019a)	Sodium acetate	Solid	Snow and ice melt	–	[Na+].[O-]C(=O)C	82.034	–

Chemical structures are represented in Simplified Molecular Input Line Entry System (SMILES) format.

In addition to the FPD, CADs contain a variety of proprietary additives designed to enhance their properties, including corrosion inhibitors, non-ionic surfactants, flame retardants, thickeners for adherence, chemicals to reduce surface tension, pH buffers, and anti-precipitation agents (Ritter, 2001; Corsi et al., 2006; Samuels et al., 2006; Adeola et al., 2009; Seeland et al., 2011). The concentrations of these additives in CADs are generally low, reportedly ranging from 1–2%v/v (Freeman et al., 2015) to 10–20%v/v (Ramakrishna and Viraraghavan, 2005). However, these additives are often difficult to remove with wastewater treatment processes (Voutsa et al., 2006; Loos et al., 2009), and thus present additional challenges for river ecology. Some additives have been shown to degrade very poorly (ACRP, 2009), persist in the environment (Breedveld et al., 2003), and even act as putative endocrine disruptors (Corsi et al., 2006). Other additives have been reported as toxic to aquatic organisms (Fisher et al., 1995; Cancilla et al., 2003; ACRP, 2009) and in isolated cases shown to decrease the biodegradation rates of FPDs such as propylene glycol (Cornell et al., 2000).

Against this background, our study aims to investigate the growth kinetics of *S. natans* in relation to URBs associated with airport anti- and de-icing activities, using controlled laboratory experiments. Specifically, our objectives are to: (i) determine which FPDs can be utilized by *S. natans*; (ii) examine differences in the growth kinetics of different FPDs; and (iii) compare pure FPDs to CADs as carbon sources, to determine impacts of additives.

It was hypothesized that: (i) ethylene glycol, propylene glycol, and sodium acetate will be utilized as carbon sources by *S. natans*, but formate cannot owing to its reportedly poor degradation by similar wastewater bacteria; (ii) lower molecular weight FPDs with higher oxygen content will promote more rapid initiation of growth leading to higher maximum growth rates (acetate > ethylene glycol > propylene glycol); (iii) CADs will have a longer lag period prior to growth, but similar bacteria growth levels, due to the reported toxicity of additives in the commercial products stunting initial substrate utilisation but not overall yield at concentrations expected in catchments. This growth data herein will help describe how airport de-icers contribute to URB growth. This information can then inform targeted treatment strategies at airports, with the ultimate goals of improving river health and quality, promoting biodiversity, and reducing anthropogenic impacts on the environment.

## 2 Materials and methods

Micro-batch culture experiments were conducted using a turbidimetric approach to monitor the growth of *S. natans* cultures using various media which incorporated different FPDs and CADs (Table 2). Optical density at 600 nm (OD_600_) of micro-batch cultures housed in 100-well microtiter plates, was recorded every 20-min over a 7-day period at a constant temperature of 25°C (high amplitude, low speed shaking ceasing 10 s prior to each measurement) (Medina Vaya et al., 2012).

**Table 2:** The carbon sources utilized in this study, along with the corresponding active ingredients in each commercial airport de-icer (CAD), and the abbreviations used to refer to each throughout this study.

Carbon source	Active ingredient	Abbreviation
Propylene glycol	–	ProG
Kilfrost DF Plus	Propylene glycol	DF
Kilfrost ABC-K Pkus	Propylene glycol	K+
Kilfrost ABC-S Plus	Propylene glycol	S+
Ethylene glycol	–	EtG
Konsin	Ethylene glycol	Kon
Sodium acetate	–	NaAce

The BioScreen C MBR (Oy Growth Curves Ab Ltd., Finland), a microtiter plate reader with controlled temperature and shaking, was used for the experiments due to its ability to measure absorbance (optical density) at selected wavelengths whilst allowing high-level control over incubation and measurement conditions and facilitating a large number of samples per run. Given the reported potential challenges of using optical density as a proxy for growth in microorganisms known to clump or form biofilms, like *S. natans* (Seder-Colomina et al., 2015). A high number of repeats were performed to minimize any effects of clumping (10 repeats and 5 blank repeats per condition were performed). The repeats were distributed across the plate to minimize discrepancies due to potential uneven heating of the microtiter plates. A strain of *S. natans* (DSM 6575) was procured from DSMZ (Leibniz Institute DSMZ GmbH, Germany). It was initially grown via incubation on its prescribed optimal growth medium (DSMZ medium 51) with beef extract (Lab Lemco, Oxoid) (5 g/L), at room temperature on a 100 rpm shaker. The culture was routinely refreshed with new media. A reference strain was used (DSM 6575), rather than an environmental isolate, to produce repeatable data with the selected strain similarly isolated from an aqueous habitat.

### 2.1 Method development

**Figure 1:**
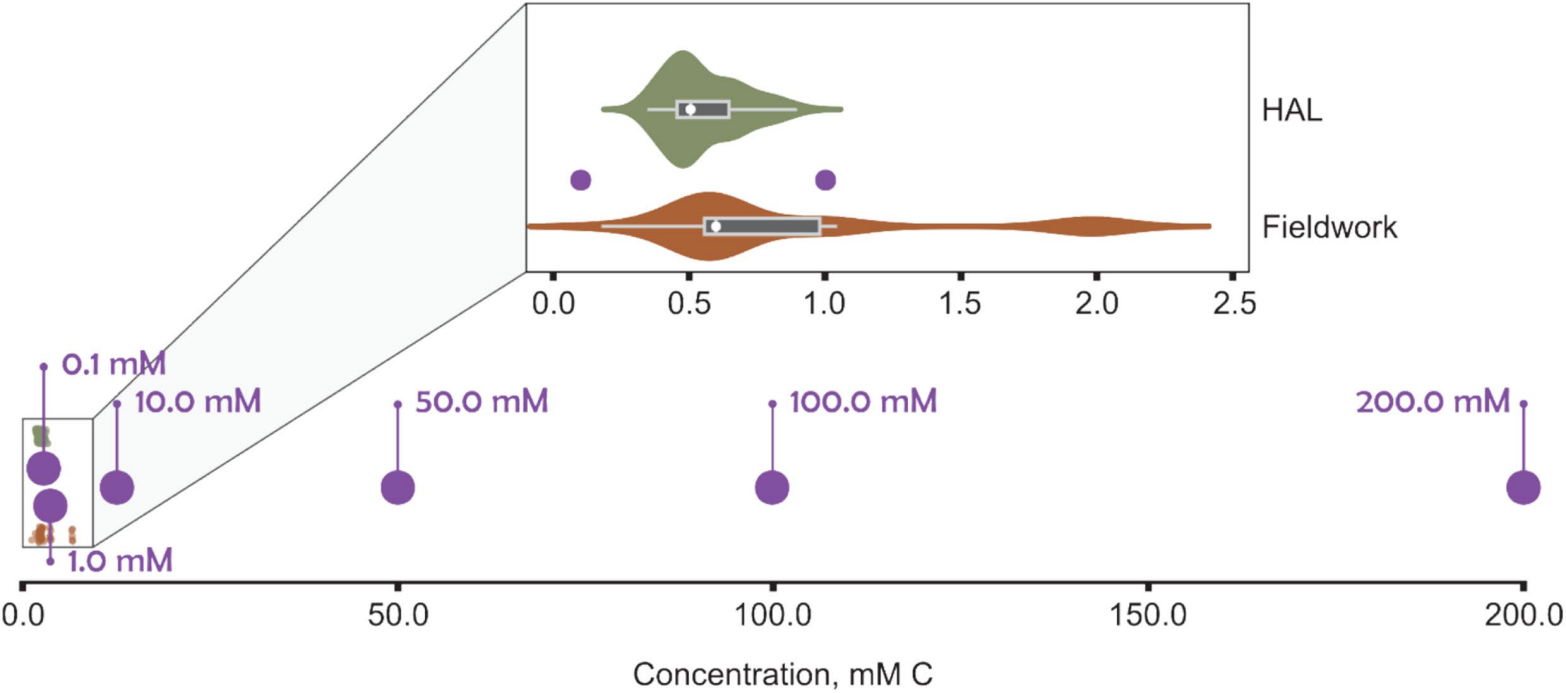
Carbon concentration in BioScreen wells (purple) compared with total organic carbon (TOC) measurements from an urban river (River Crane, London; TQ 10958 75,226) receiving airport runoff recorded by the authors (“Fieldwork,” orange), in addition to monthly monitoring samples of the river by contractors on behalf of Heathrow Airport Ltd. (“HAL,” green).

The River Crane (London, UK) serves as an example catchment that receives treated surface water effluents from airport de-icing (Exton et al., 2023). It is a highly urbanized catchment with lots of pressures on the riverine ecosystem. During preliminary experiments, a range of peak FPD concentrations from the River Crane were initially used, as reported by the Environment Agency (UK environment regulator), a field study by the authors, and monthly sampling on behalf of Heathrow Airport Ltd. Data from the Environment Agency’s Water Quality Archive indicated that most monthly measurements of total glycols in the River Crane were below 0.00001 mg/L (Environment Agency, and Department for Environment Food & Rural Affairs, 2020). However, occasional surges of glycol concentrations in winter and early spring ranged from 1.06 to 64.60 mg/L (approximately 0.014–1.041 mM C). In addition, sampling in the River Crane by the authors (Exton et al., 2023) – conducted over two winter seasons (October, 2020 to April 2022), downstream of airport outfalls, and including an URB event – reported total organic carbon (TOC) values primarily between 0.2 and 2.0 mM C (Figure 1). Furthermore, monthly spot sampling by contractors on behalf of Heathrow Airport Ltd. recorded TOC between 0.35–0.90 mM C. These three datasets corroborated each other, providing similar ranges of total glycols or total organic carbon and gave a consistent basis for our experimental design.

Initial experiments using environmentally relevant carbon concentrations (0.1 and 1.0 mM C of FPD) did not stimulate sufficient growth of *S. natans* to produce a discernible ΔOD_600_. This lack of detectable growth may reflect the sensitivity of the instrument but is likely fundamentally due to nutrient, specifically carbon, limitation in the fixed micro-batch cultures. URB growth requires flowing water to replenish essential nutrients such as carbon, nitrogen, phosphorus, and dissolved oxygen (Curtis, 1969; Battin et al., 2007) and, as such, even lower organic carbon loading can promote substantial growth in flowing waters (Curtis et al., 1971).

Theoretical calculations, using measured carbon concentrations (Figure 1) and discharge data (National River Flow Archive, and UK Centre for Ecology and Hydrology, 2023) from the River Crane were performed to understand the volumetric and chemical flux of organic carbon. Calculations also used the time required for propylene glycol, ethylene glycol, and sodium acetate cultures to reach maximum OD_600_ (60, 84, and 48 h respectively). Over these durations, the equivalent carbon concentration URBs would be exposed to is 4.8 × 10^7^–30.0 × 10^7^ mmol C. These amounts of carbon are many orders of magnitude greater than the amount used in micro-batch cultures (2 × 10^−5^ – 4 × 10^−2^ mmol C for 200 μL of 0.1–200 mM C). Therefore, even assuming most organic carbon in the river is not assimilable, flowing waters with substantially lower TOC concentrations than used in fixed micro-batches here still have sufficient organic carbon to promote URB growth.

The fixed micro-batch cultures and absorbance-based approach present a limitation of this research, compared to directly evaluating the growth of fixed polymicrobial biofilms, as found in rivers. However, in studying these tightly controlled micro-batch cultures, a wide range of treatments could be tested and compared. Therefore, a higher nutrient concentration than found supporting URB growth in rivers was required in these experiments. Furthermore, we needed to work within the detection limits of the instrument (0.0–2.0 abs) (Oy Growth Curves Ab Ltd, 2017).

Other essential nutrients, such as nitrogen and phosphorus, are described as sufficiently abundant in natural freshwaters so as to not limit URB growth (Curtis and Harrington, 1971; Curtis et al., 1971). In the minimal media used here, nitrogen sources made up 20 mM N and phosphorus sources make up 6.7 mM P. Concentrations of these nutrients measured in the River Crane during fieldwork by the authors were 0.17–0.49 mM NO_3_-N and 0.005–0.012 mM PO_4_-P, several orders of magnitude below that used in experimental media.

Additional pilot experiments established that a starting OD_600_ equivalent to 0.005 (measured at 0.5 and diluted 1:100) in micro-batch cultures allowed clear and distinct growth profiles to be observed over time within the operational range of the BioScreen C MBR (0.0–2.0 abs) (Oy Growth Curves Ab Ltd, 2017).

### 2.2 Media selection and preparation

To meet the objectives of this study, several pure-FPDs and CADs at concentrations of 0.1, 1.0, 10, 50, 100, and 200 mM C were tested (Table 2). Given that assimilable organic carbon is the main driver of URB growth, de-icer concentrations were standardized based on the carbon concentration – in terms of mmol/L of carbon (mM C). Another approach to standardize carbon concentrations could have been to use carbon-derived reducing equivalents (Appendix 1), however, moles of carbon was chosen because it is the limiting nutrient and the driver of microbial growth in URB taxa, as well as a more commonly used metric in microbial growth experiments. Concentrations ≥10 mM C were sufficient to promote discernible growth but not exceed the maximum OD_600_ absorbance of the instrument (Abs ≤2.0). It should be noted that the exact concentrations of FPDs in different CAD products are not universally reported. As such, FPD concentrations were determined using High-performance liquid chromatography (HPLC, Agilent Technologies 1200 series, USA) with specific settings (10.0 μL injection volume, 0.400 mL/min flow rate, 21–24 bar, 60°C), column (ROA Rezex Phenomenex 150 × 7.8 mm column, refractive index detector), and mobile phase (H_2_SO_4_, 0.5 mM). Stock solutions of FPDs/CADs were prepared in a sterile environment. The solutions had double the final concentrations and were prepared in a carbon-free minimum essential media (MEM) with sufficient nitrogen and phosphorus so as to not limit growth, even at the highest carbon concentrations tested. This MEM was based on the M9 minimal medium with calcium added to better support *S. natans* growth (Dias et al., 1968) using: ATCC^®^ MD-*VS*™ (10 mL/L); ATCC^®^ MD-TMS™ (10 mL/L), Na_2_HPO_4_ (6.8 g/L), KH_2_PO_4_ (3.0 g/L), NH_4_Cl (1.0 g/L); and CaCl_2_ (11.098 mg/L). Once prepared, the FPD/CAD stock solutions were refrigerated (4°C) until use. Aliquots of FPD and CAD stock solutions were dispersed in 100-well sterile BioScreen C MBR plates (100 μL/well). Repeats of each FPD/CAD at their various concentrations were distributed across the plate to reduce errors attributed to unequal heating. A sample of the *S. natans* culture was then taken and diluted to achieve an OD_600_ of 0.005 in MEM. Following the setup, both microtiter plates were placed into the BioScreen C MBR, and the pre-configured settings were applied.

### 2.3 Data analysis and statistics

Data analysis involved the baseline correction of OD_600_ measurements based on the average OD_600_ of 40–100 min (i.e., the average for the first hour after readings were allowed to initially settle) of each individual sample. A calculated LOD using baseline corrected optical density of microorganism-negative blanks was found to be 0.017. Averages and confidence intervals of all repeats were then calculated for line graphs. Three indicators of growth kinetics were calculated: time-to-detection (TTD), maximum rate of change (maxROC), and maximum optical density at 600 nm (maxOD). TTD, the time taken for the sample to reach an OD_600_ of 0.1 (after baseline correction), indicates the delay in the utilisation of the carbon source. TTD is a direct assessment of the lag phase duration (“lag-time”) by setting an arbitrary OD_600_ achieved to indicate the start of the exponential phase of microbial growth (Medina Vaya et al., 2012; Baka et al., 2015; Danial et al., 2020). An absorbance of 0.1 was chosen following a visual inspection of growth curves to determine the start of exponential growth in relation to the maxOD achieved in these media for this starting cell concentration of this specific taxon and sufficiently above the calculated limit of detection (LOD = 0.0173) to avoid false positives. LOD was calculated based on the mean plus three standard deviations of the baseline-corrected OD data for “blanks” (i.e., containing no *S. natans*). Secondly, maxROC indicates the highest linear rate of ΔOD_600_ in the exponential growth phase (Aguirre et al., 2014) and was calculated by taking the maximum gradient calculated over 3 h of 1-h moving average values (“growth rate”) (Zwietering et al., 1990). Moving averages and a 3-h gradient were used to smooth out sudden fluctuations in the OD_600_ seen between individual measurements – a limitation with using optical density measurements, especially in taxa that clump or form flocs (Seder-Colomina et al., 2015). Finally, maxOD indicates the maximum turbidity of each sample, calculated simply by finding the maximum corrected OD_600_ of each sample, as a proxy for the highest bacterial load the specific medium can support (“total growth”). These kinetic indicators allowed for direct comparison between different media at varying concentrations and statistical analyses to be performed. The Kruskal–Wallis non-parametric test of difference and pairwise comparisons were used, as the assumptions of ANOVA (normality) were not met.

## 3 Results and discussion

**Figure 2:**
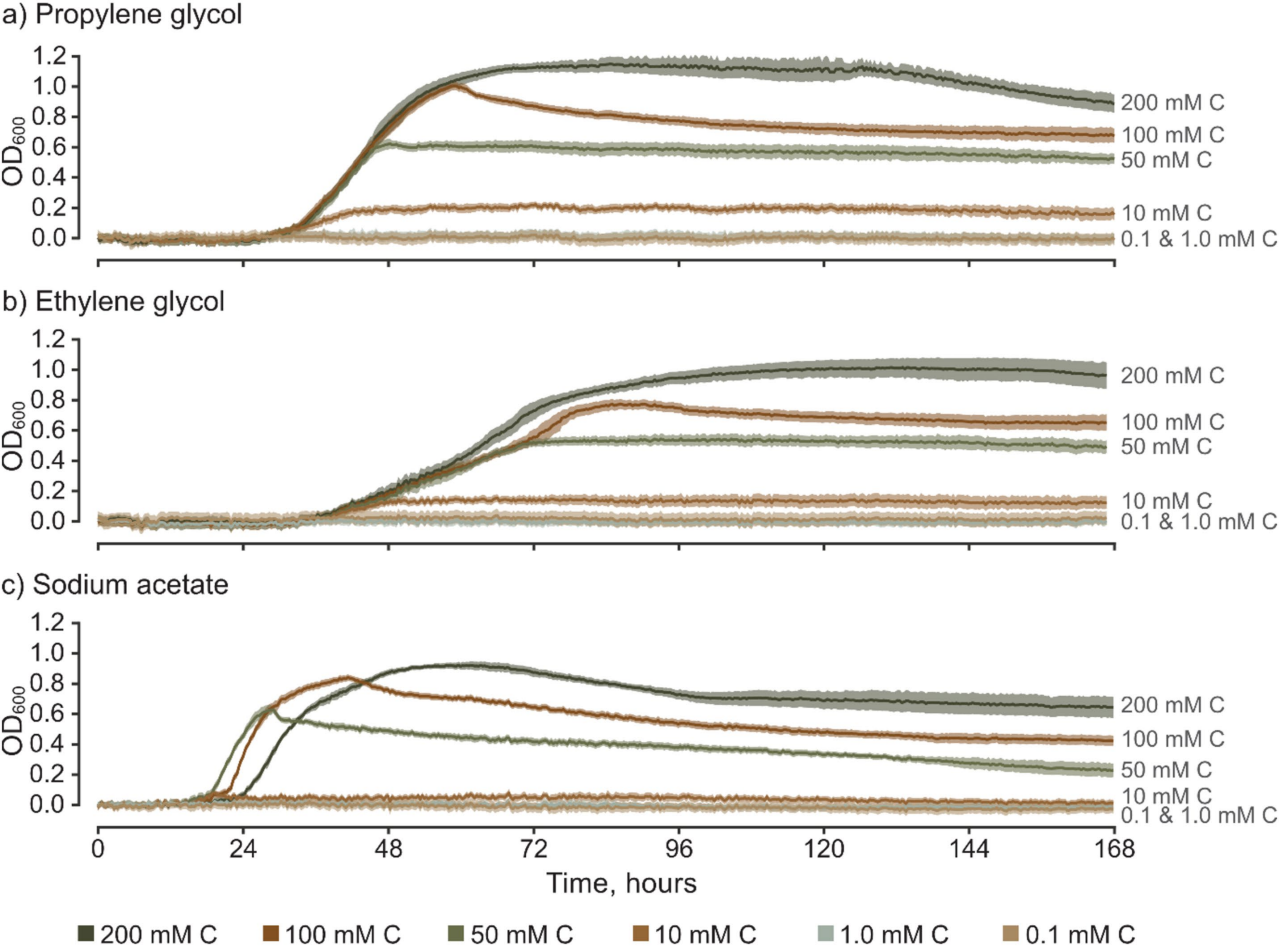
Time-series of optical density for *S. natans* culture grown in media based on various freeze-point depressants, presented across a range of carbon concentrations (mM C). Error bars represent 95% confidence intervals (*z* = 1.96) based on 10 replicates for each treatment. Graphs show results for **(a)** propylene glycol, **(b)** ethylene glycol, and **(c)** sodium acetate.

The recorded changes in OD_600_ (ΔOD_600_) show that *S. natans* can utilize propylene glycol, ethylene glycol, and sodium acetate as primary carbon sources (Figure 2). This substantiates, for the first time and under controlled conditions, a direct correlation between FPD-contaminated discharges and the support or facilitation of URBs growth. At concentrations equal to or above 10.0 mM C, the OD_600_/time growth curves showcase sigmoidal (“S-shaped”) growth, which is characteristic of microbial growth in micro-batch culture experiments without significant carbon inhibition. These patterns are consistently similar across various FPDs, albeit with minor differences observed between them. For example with sodium acetate, where mean OD_600_ measurements for 10 mM C cultures were largely within the 95% confidence intervals for 0.1 and 1.0 mM C which were concluded to be insufficient to promote *S. natans* growth. Overall, there is clear positive correlation between total growth and carbon concentration, with notable differences between carbon sources which underscores the importance of these assailable carbon sources as primary drivers of *S. natans* growth and limiters at low C. Nevertheless, *S. natans* growth was not stimulated by either sodium formate or potassium formate, which are commonly used as ground de-icers. To substantiate these findings and validate the viability of the culture-based approach, experiments with formate salts were repeated, including wells with other inoculated FPDs and ideal medium (medium 51, DSMZ). Consistently, *S. natans* showed no detectable increase in OD_600_ in the presence of formate salts, yet robust growth was observed in wells containing all other carbon sources. It should be noted, however, that the inability of *S. natans* to utilize formate salts as a carbon source does not imply that URBs, as a whole, share this limitation. Given the complex assemblage of microorganisms that constitute URBs, formate salts may still be bioavailable to other taxa within these complex polybacterial biofilms, such as certain *Zoogloea* spp. (Unz and Dondero, 1967a, 1967b). Moreover, the presence of specific nutrients, like formate, can potentially affect the microbial composition of URBs, thereby optimizing the biofilm’s ability to metabolize available nutrients (Battin et al., 2016).

### 3.1 FPD active ingredients and concentrations on *Sphaerotilus natans* growth

**Figure 3:**
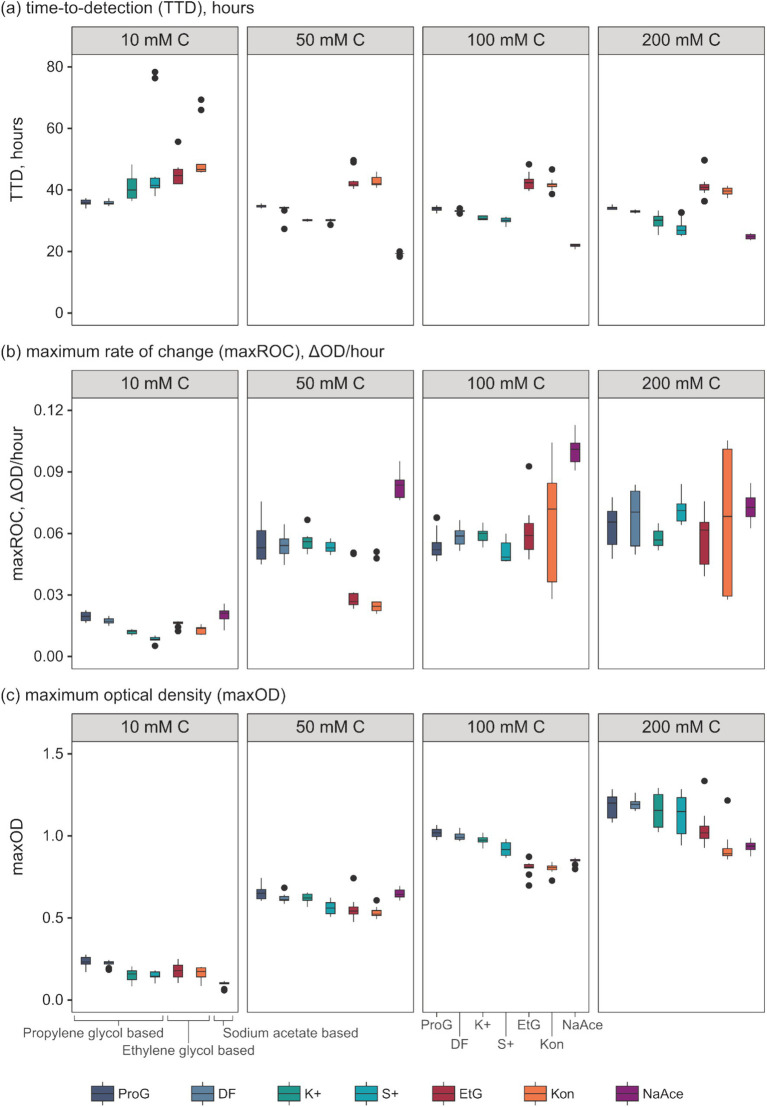
Evaluation of growth kinetic parameters of *S. natans* in response to different pure freeze-point depressants (FPDs) and commercial airport de-icers (CADs) across various concentrations. Presented parameters include **(a)** time-to-detection (TTD, “lag phase”), **(b)** maximum rate of change (maxROC, “growth rate”), and **(c)** maximum optical density (maxOD, “total growth”). The utilised carbon sources include propylene glycol (ProG), Kilfrost DF Plus (DF), Kilfrost ABC-K Plus (K+), Kilfrost ABC-S Plus (S+), ethylene glycol (EtG), Konsin (Kon), and sodium acetate (NaAce). Box plots depict the interquartile ranges, and whiskers represent the ranges, extending up to 1.5 times the interquartile range based on 10 replicates for each treatment.

Analysis of the growth kinetics identified significant differences in TTC, maxROC, and maxOD between FPDs and CADs, which varied by carbon concentration (Figure 3). The TTD varied depending on the type of FPD used (Figure 3a). Ethylene glycol had a significantly longer TTD (averaging 41.2 to 44.2 h) compared to propylene glycol (33.9–35.9 h) and sodium acetate (19.2–24.8 h) (*p* = 0.000–0.028), which had a notably shorter TTD than either glycol-based FPD (p = 0.000–0.003) (Table 3). These variations in TTD could point to differences in the bioavailability of each carbon source for *S. natans*. The longer TTD for ethylene glycol implies that *S. natans* takes more time to initiate growth in this medium, suggesting that it may be less readily utilized by the organism. Conversely, the shorter TTD for sodium acetate indicates that *S. natans* can begin using this compound more quickly, implying it might be more easily assimilated as a carbon source. Therefore, TTD can be seen as a reflection of the lag phase of growth, demonstrating the time taken by the organism to adapt to the medium and initiate exponential growth. The extended TTD for ethylene glycol was unexpected, given its elemental structure and molecular mass closely resemble the acetate anion, and it has a lower molecular weight, more oxygen-rich structure compared to propylene glycol. These TTD are substantially longer than the <24 h that *S. natans* took to reach a maxOD in its ideal medium (beef broth, DSMZ medium 51) (Leibniz Institute DSMZ GmbH, Germany) (Appendix 1).

**Table 3:** Pairwise comparisons from Kruskal–Wallis test of difference of the time-to-detection (TTD), maximum rate of change (maxROC) and maximum optical density (maxOD) between freeze-point depressants [propylene glycol (ProG), ethylene glycol (EtG), and sodium acetate (NaAce)] at the concentrations **(a)** 200 mM C, **(b)** 100 mM C, **(c)** 50 mM C, and **(d)** 10 mM C.

**(a) 200 mM C**									
	**TTD**			**maxROC**			**maxOD**		
	ProG	EtG	NaAce	ProG	EtG	NaAce	ProG	EtG	NaAce
ProG	–			–			–		
EtG	0.028*	–		0.472	–		0.411	–	
NaAce	0.003*	<0.001*	–	0.264	0.066	–	0.060	0.290	–

(b) 100 mM C									
	TTD			maxROC			maxOD		
	ProG	EtG	NaAce	ProG	EtG	NaAce	ProG	EtG	NaAce
ProG	–			–			–		
EtG	0.005*	–		0.407	–		0.039*	–	
NaAce	0.002*	<0.001*	–	0.001*	0.009*	–	0.073	0.787	–

(c) 50 mM C									
	TTD			maxROC			maxOD		
	ProG	EtG	NaAce	ProG	EtG	NaAce	ProG	EtG	NaAce
ProG	–			–			–		
EtG	0.022*	–		0.074	–		0.364	–	
NaAce	<0.001*	<0.001*	–	0.006*	<0.001*	–	0.982	0.352	–

(d) 10 mM C									
	TTD			maxROC			maxOD		
	ProG	EtG	NaAce	ProG	EtG	NaAce	ProG	EtG	NaAce
ProG	–			–			–		
EtG	0.017*	–		0.642	–		0.559	–	
NaAce	–	–	–	0.982	0.626	–	0.164	0.418	–

Where * indicates significant difference (*p* = 0.05).

Whilst the FPD concentration exerts a considerable influence on the overall growth pattern of *S. natans*, it appears to exert minimal impact on the TTD (Figure 3a and Table 4). No significant variations were observed in the TTD across different concentrations (≥10 mM C) of each FPD (*p* ≥ 0.306). However, it is worth noting that at a concentration of 10 mM C, a larger range of TTD values is observed compared to higher concentrations suggesting that whilst 10 mM C is the minimum concentration that facilitates discernible growth, it may also serve as a limiting factor for *S. natans*. Furthermore, sodium acetate displayed a smaller standard deviation (0.45–0.85 h) across repetitions, indicating a lesser variance in TTD. Which is particularly noteworthy when compared to other carbon sources, such as propylene and ethylene glycol, which had much larger standard deviations (0.50–1.06 and 2.80–4.23 h, respectively; Figure 3a). This data suggests that sodium acetate may provide a more consistent substrate for the growth of *S. natans.* Sodium acetate’s suitability as a superior growth substrate for *S. natans* could stem from its water solubility, facilitating easier assimilation, and its chemical structure, favoring metabolic incorporation. Its high solubility could allow acetate to be readily available in the environment for bacterial uptake. Additionally, as a small two-carbon compound, acetate might be more easily processed by *S. natans*. Importantly, acetate can directly feed into the Krebs cycle, a central metabolic pathway for energy production. Acetate is converted into acetyl-CoA, a key intermediate in the cycle. This efficient utilisation might explain the shorter TTD with sodium acetate, suggesting quicker growth initiation. Propylene and ethylene glycol, despite their similar two and three-carbon structures respectively, do not exhibit the same uptake by *S. natans*. These differences might be because their metabolic incorporation involves a more complex process, likely passing through the glycolytic pathway, a less direct route compared to acetate. Additionally, their larger molecular size might also contribute to slower assimilation rates, as observed by the longer TTD.

**Table 4:** Pairwise comparisons using the Kruskal–Wallis test to analyze differences in time-to-detection (TTD), maximum rate of change (maxROC), and maximum optical density (maxOD) between concentrations (mM C) of the freeze-point depressants **(a)** propylene glycol, **(b)** ethylene glycol, and **(c)** sodium acetate.

(a) Propylene glycol												
	TTD				maxROC				maxOD			
	10.0	50.0	100.0	200.0	10.0	50.0	100.0	200.0	10.0	50.0	100.0	200.0
10.0	–				–				–			
50.0	0.671	–			0.007*	–			0.074	–		
100.0	0.306	0.549	–		0.012*	0.857	–		<0.001*	0.004*	–	
200.0	0.403	0.681	0.851	-	<0.001*	0.307	0.230	-	<0.001*	<0.001*	0.372	-

(b) Ethylene glycol												
	TTD				maxROC				maxOD			
	10.0	50.0	100.0	200.0	10.0	50.0	100.0	200.0	10.0	50.0	100.0	200.0
10.0	–				–				–			
50.0	0.604	–			0.167	–			0.144	–		
100.0	0.555	0.942	–		<0.001*	0.015*	–		0.001*	0.082	–	
200.0	0.307	0.615	0.666	–	0.001*	0.037*	0.729	–	<0.001*	<0.001*	0.033*	–

(c) Sodium acetate												
	TTD				maxROC				maxOD			
	10	50	100	200	10	50	100	200	10	50	100	200
10	–				–				–			
50	–	–			<0.001*	–			0.001*	–		
100	–	0.773	–		<0.001*	0.603	–		<0.001*	0.281	–	
200	–	0.536	0.741	–	<0.001*	0.554	0.266	–	<0.001*	0.060	0.421	–

Where * indicates significant difference (*p* = 0.05).

**Figure 4:**
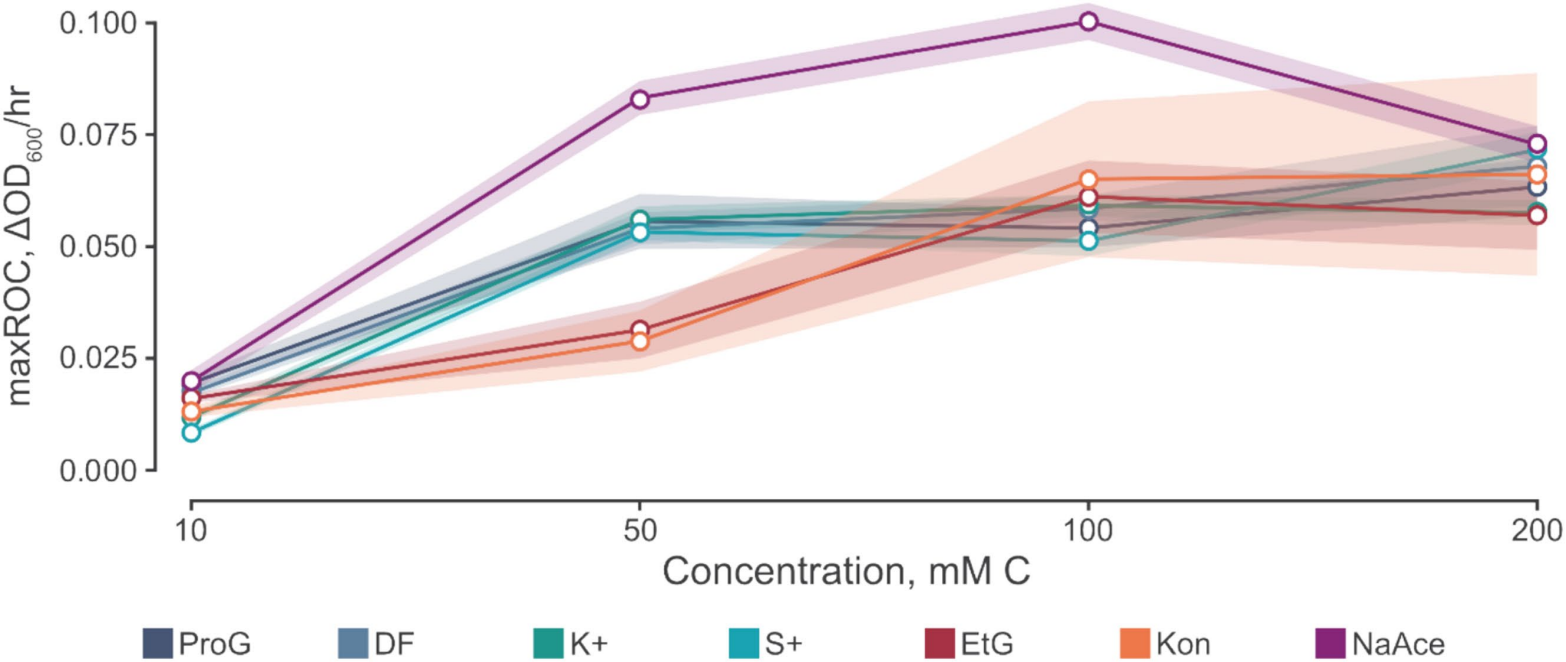
The relationship between maximum rate of change (maxROC, ΔOD_600_/h) and concentration of carbon source. Carbon sources tested include propylene glycol (ProG), Kilfrost DF Plus (DF), Kilfrost ABC-K Plus (K+), Kilfrost ABC-S Plus (S+), ethylene glycol (EtG), Konsin (Kon), and sodium acetate (NaAce). Error is denoted by mean ± confidence intervals (*z* = 1.96) based on 10 biological replicates for *S. natans* cultures.

The data presented in Table 4 implies a concentration threshold for FPDs that significantly impacts microbial growth, most visible in the maxROC (Figure 4). For treatments utilizing propylene glycol and sodium acetate, a distinct variation is observed between 10 mM C and the higher concentrations (50, 100, and 200 mM C) (*p* ≤ 0.012, Tables 4a,c), implying a limiting concentration between 10 and 50 mM C for these carbon sources (Figure 3b). For ethylene glycol, the notable change in maxROC occurs between 50 and 100 mM C (*p* = 0.015, Table 4b), suggesting a higher growth limit concentration. Above these threshold concentrations, the variations in maxROC between concentrations appear consistent for both propylene and ethylene glycol (*p* = 0.203–0.857, Table 4a; *p* = 0.729, Table 4b respectively).

Interestingly, sodium acetate often exhibits a higher maxROC compared to propylene and ethylene glycol, except at limiting concentrations of 10 mM C and at 200 mM C where the maxROC drops (Figure 3b and Table 3). The decline in maxROC between 100 and 200 mM C of sodium acetate is not significant (*p* = 0.266), but it may hint towards osmotic stress, toxicity at elevated concentrations, or metabolic burden. Further research is needed to determine the mechanisms underlying these results.

At elevated substrate concentrations, the suppression of growth (as indicated by the reduction in maxROC) can occur due to several factors. A primary reason could be osmotic stress. High concentrations of solutes in the medium can cause water to move out of the microbial cells to balance the osmotic pressure, leading to dehydration and slower growth. Another factor could be substrate toxicity (Okpokwasili and Nweke, 2006). Even essential nutrients can become toxic at high concentrations. The FPDs may interfere with cellular mechanisms, enzymatic reactions, or membrane integrity at elevated levels, causing a decrease in cell viability and consequently growth. In addition, metabolic burden or overflow metabolism might come into play. When substrate availability is high, cells could take up the substrate at a rate exceeding the capacity of the downstream metabolic pathways. Which may lead to the accumulation of metabolic intermediates or by-products that are toxic to the cells, thereby inhibiting growth. Among the FPDs, the effect of high substrate concentration might be more pronounced for those that are metabolized more slowly or those that have more potential for toxicity at high levels. For instance, ethylene glycol, which is metabolized in several steps involving conversion to toxic intermediates like glycolic acid and oxalic acid, could show a greater suppressive effect at high concentrations compared to others (Carney et al., 1999).

As expected, maxOD escalated with carbon concentration (Figure 3c), leading to significant differences between each concentration for all FPDs (Table 3). These differences indicate the strong dependency of *S. natans* and URBs’ on organic carbon. Propylene glycol consistently induced a slightly higher maxOD than other carbon sources (Figure 3c), but with no statistically significant difference (Table 3), except against ethylene glycol at 100 mM C (*p* = 0.039). The comparable maxOD between FPDs was anticipated, as concentration was standardized by moles of carbon to facilitate direct comparison across FPDs.

Another approach to compare FPDs is by using carbon-derived reducing equivalents (Appendix 1). Applying this method indicates there was a higher maxOD per reducing equivalent for acetate, then ethylene glycol, and then propylene glycol (Appendix 2); meaning we would expect more URB growth per reducing equivalent for acetate-based CADs. These findings contrast evaluations based on growth per moles of FPD or per mass of FPD which reveals higher maxOD per mole or gram of FPD using propylene glycol, followed by ethylene glycol and then acetate (Appendix 2). Therefore, these approaches suggest that there would be more URB growth per mM or mg/L of propylene glycol contamination in outfalls. When evaluating microbial growth by moles of carbon (mM C), the negligible differences between FPDs are attributed to the minor variations in average maxOD. These variations were found to be statistically insignificant, except between propylene glycol and ethylene glycol at 100 mM C (Table 3). Each of these metrics – carbon-derived reducing equivalents, mass concentration of FPD, molar concentration of FPD, and molar concentration of carbon – provides a valid perspective for interpreting the growth of *S. natans*. Environmental monitoring typically uses the mass concentration of a contaminant (e.g., mg/L of propylene glycol); however, in this study, we standardized the concentration by moles of carbon, because carbon is the primary driver of URB growth, is a more direct approach, and is a more commonly used parameter than carbon-derived reducing equivalents.

In conclusion, there were statistically significant differences in the growth kinetics of propylene glycol, ethylene glycol, and sodium acetate. The major findings were (i) sodium acetate had a faster TTD and higher maxROC than propylene glycol and finally ethylene glycol; (ii) the influence of a limiting concentration on growth kinetics (10 mM C for propylene glycol and sodium acetate, 50 mM for ethylene glycol), specifically on maxROC; and (iii) maxOD was higher with propylene glycol.

### 3.2 Analysis of pure FPDs and corresponding CADs

**Figure 5:**
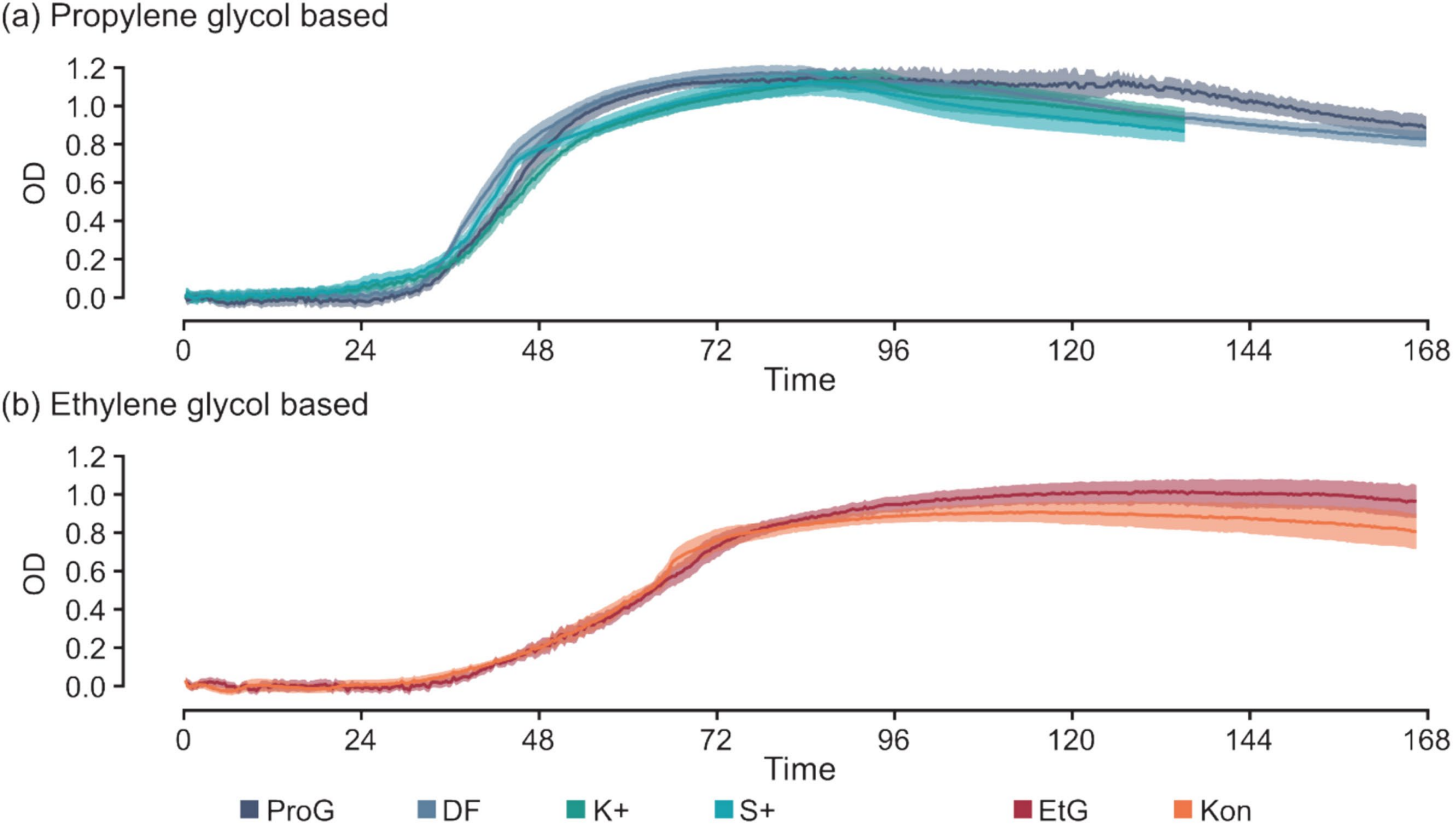
Time-series of optical density for *S. natans* culture between pure freeze-point depressants (FPDs) and their commercial products (CADs) at 200 mM C for *S. natans* cultures in **(a)** propylene glycol based and **(b)** ethylene glycol based media. Error is denoted by mean ± confidence intervals (*z* = 1.96) based on 10 biological replicates for *S. natans* cultures. Carbon sources include propylene glycol (ProG), Kilfrost DF Plus (DF), Kilfrost ABC-K Plus (K+), Kilfrost ABC-S Plus (S+), ethylene glycol (EtG), Konsin (Kon), and sodium acetate (NaAce).

Additives in CADs exhibit minimal effects on *S. natans* growth (Figure 5). Minor variations are noticeable in select parameters, for example, the TTD between K+ and ProG at 50 mM C (*p* = 0.016), and S+ and ProG at 50 and 200 mM C (*p* = 0.019 and 0.017) (Figure 3a and Table 5). These slight differences suggest that the impact of additives on growth, if any, is minor, and does not significantly alter the detection time. The similarity in maxROC and maxOD between FPD and CAD further substantiates that commercial additives do not significantly influence *S. natans* growth kinetics (Figures 3b,c and Table 5). The marginal impact of additives may be due to the low concentration used in CAD formulations, further diluted in runoff and river water, and thus may not sufficiently interact or interfere with *S. natans* to influence growth, even if toxic to other aquatic fauna and flora (Hartwell et al., 1995; Breedveld et al., 2003; Cancilla et al., 2003; Corsi et al., 2006; Seeland et al., 2011). Additionally, these additives might not be metabolically usable by *S. natans* or their impact is overshadowed by the more abundant FPDs.

**Table 5:** Pairwise comparisons from Kruskal–Wallis test of difference of the time-to-detection (TTD), maximum rate of change (maxROC) and maximum optical density (maxOD) between freeze-point depressants and their commercial product at the concentrations **(a)** 200 mM C, **(b)** 100 mM C, **(c)** 50 mM C, and **(d)** 10 mM C.

**(a) 200 mM C**												
	**TTD**				**maxROC**				**maxOD**			
	DF	K+	S+	Kon	DF	K+	S+	Kon	DF	K+	S+	Kon
ProG	0.417	0.071	0.017*		0.772	0.570	0.306		0.987	0.883	0.701	
EtG				0.615				0.655				0.268

(b) 100 mM C												
	**TTD**				**maxROC**				**maxOD**			
	DF	K+	S+	Kon	DF	K+	S+	Kon	DF	K+	S+	Kon
ProG	0.618	0.178	0.081		0.448	0.373	0.660		0.813	0.561	0.274	
EtG				0.904				0.630				0.930

(c) 50 mM C												
	**TTD**				**maxROC**				**maxOD**			
	DF	K+	S+	Kon	DF	K+	S+	Kon	DF	K+	S+	Kon
ProG	0.525	0.016*	0.019*		0.889	0.861	0.792		0.747	0.787	0.389	
EtG				0.933				0.826				0.845

(d) 10 mM C												
	TTD				maxROC				maxOD			
	DF	K+	S+	Kon	DF	K+	S+	Kon	DF	K+	S+	Kon
ProG	0.962	0.174	0.080		0.779	0.317	0.169		0.958	0.389	0.376	
EtG				0.682				0.690				0.886

Carbon sources include propylene glycol (ProG) and its commercial products (CADs) Kilfrost DF Plus (DF), Kilfrost ABC-K Plus (K+), Kilfrost ABC-S Plus (S+) and ethylene glycol (EtG) and the CAD Konsin (Kon). Where * indicates significant difference (*P* = 0.05).

Existing literature provides limited information on the specific chemicals used as additives in CADs (ACRP, 2009), although reports frequently note their toxic effects (Cancilla et al., 2003; Corsi et al., 2006). Additives such as benzotriazoles, commonly included in CAD formulas as corrosion inhibitors, are known for their acute and chronic toxicities to various aquatic species (Breedveld et al., 2003; Seeland et al., 2011) including the fathead minnow (Hartwell et al., 1995). As a result, manufacturers are reportedly reducing the quantity of benzotriazoles in their CADs (Olds et al., 2021). Other additives include alkylphenol ethoxylates, which serve to reduce surface tension but are also reported to be toxic to aquatic organisms, degrading into by-products identified as endocrine disruptors (Corsi et al., 2006). However, not all additives have harmful effects; for example, phosphate salts are used as benign pH buffers (Samuels et al., 2006). Previous studies predominantly focus on the toxicity of these compounds to fish and invertebrates. The current research indicates that the additives in CADs exert negligible impact on *S. natans* growth. Nonetheless, when organic pollution ceases and URBs decompose, any remaining undegraded compounds are released back into the water system, potentially causing further ecological deterioration. Thus, a holistic understanding of the impact of these additives on various elements of aquatic ecosystems is essential for sustainable management.

### 3.3 New insights in URB growth from high-frequency measurements

In this research, a turbidimetric micro-batch cultures design was deployed to evaluate the growth of URB taxa, using airport de-icers as the sole carbon source. The study revealed that FPDs such as propylene glycol, ethylene glycol, and sodium acetate, can stimulate *S. natans* growth. Moreover, no substantial differences were observed between the growth induced by pure FPDs and CADs.

Carbon concentrations below 10.0 mM C failed to stimulate growth, despite observed TOC in a case study river (River Crane, London) receiving treated airport surface runoff not surpassing 2.0 mM C. However, in flowing water bodies, nutrients like organic carbon are continuously replenished (Phaup, 1968; Curtis, 1969; Quinn and Mcfarlane, 1985; Gray, 1987), necessitating lower background nutrient concentrations. Hence, in fixed micro-batch cultures, higher initial nutrient concentrations are required.

The turbidimetric micro-batch culture method used in this study enabled comparison of a large number of FPDs and CADs in an efficient and cost-effectiveness manner. ‘As previous research has shown that turbidimetric approaches might lead to under- or overestimation of cell numbers (Seder-Colomina et al., 2015), we used a large number of replicates (n = 10) capture variance potentially caused by cell clumping.

Future research could expand on this study’s findings, exploring the growth of other URB taxa and extending the focus to mixed species cultures that more accurately represent polymicrobial biofilms. Methods such as time-lapse microscopy (e.g., oCelloScope by BioSense Solutions ApS, Denmark) could help understand interactions between taxa and relative growth rates whilst retaining the benefits of optical density measures (Zhang et al., 2019). These methods would be beneficially complemented by larger, mesocosm experiments such as flumes, to investigate the effects of flow rate, background water quality, and mixed cultures on URB formation and growth (Toprak et al., 2012).

This work significantly contributes to our understanding of how airport de-icing compounds, both pure and in commercial formulations, influence the growth of ubiquitous river bacteria, *S. natans*. It elucidates key growth parameters such as TTD, maxROC, and maxOD across various concentrations of FPDs, offering valuable insights into the microbial response to these compounds. This research uniquely demonstrates that the additives within commercial de-icers have minimal impact on *S. natans’* growth kinetics, providing a more nuanced understanding of these agents’ ecological impacts. Additionally, the findings challenge preconceived notions about the toxicity of these compounds to URBs, in which *S. natans* is a dominant component (Curtis, 1969; Geatches et al., 2014; Nott et al., 2020; Exton et al., 2023), and highlight the necessity for a more comprehensive understanding of the environmental implications of de-icing chemicals, particularly in light of changing climatic conditions. As such, this study forms a solid basis for future research on microbial responses to pollutants in aquatic environments. Although this research should still examine the influence of CAD additives on microbial growth, compared to pure-FPDs.

The study offers valuable insights into the impacts of commonly used airport de-icers on river bacteria for a range of stakeholders. Regulatory bodies and policymakers concerned with environmental health and water quality can leverage these findings to drive environmentally-conscious policy development. Furthermore, airport operators, particularly those involved in operations and environmental compliance, are key beneficiaries. For example, understanding the influences of different FPDs and additives on microbial growth could influence procurement decisions and waste management practices, potentially leading to more sustainable airport operations. In turn influencing de-icer manufacturers to optimize their products, reduce environmental impact whilst maintaining efficacy. Finally, this study reinforces the importance of robust, science-based decision-making in industries that interface directly with our natural ecosystems.

This study examined the growth dynamics of suspended cultures of a characteristic microorganism within a type of biofilm (i.e., URBs), which is not truly representative of their growth in natural ecosystems presenting a limitation of this study. Furthermore, the constraints of operating absorbance detection limits (0.0–2.0 abs), fixed and non-replenishable source of nutrients, in addition to microcosm volume constraints present compromises made to facilitate the high-replication and wide range of unique treatments as an initial investigation into CAD contamination in surface water discharges and the subsequent growth of URBs. Alternative methodologies, such as microfluidic devices (Kumar et al., 2012; Karimi et al., 2015; Liu et al., 2019) or flow cells with microscopy (Tolker-Nielsen and Sternberg, 2014), offer opportunities to study microbial growth within biofilms, thus with greater ecological relevance. Moreover, these alternative approaches could be performed at temperatures more representative of the environment URBs grow in, rather than the standard ‘lab temperature’ used here. Another approach worthy of investigation is using larger-scale experimental design, even more representative of the environmental system it seeks to emulate, such as flumes or recirculating channels (Curtis et al., 1971) Such larger-scale experiments would also enable the quantification of nutrients in the matrix and their consumption overtime in relation to microbial growth but such designs have the trade-off that a reduced number of treatments can be tested. Whilst these methods provide valuable insights into biofilm formation and microbial interactions, studying a diverse range of treatments and environmental conditions that affect microbial growth is more challenging than using highly robust optical density methods. This study presents a novel dataset demonstrating the ability of *S. natans* to utilize FPDs for growth, solidifying its involvement in airport de-icer implicated URBs. Whilst directly measuring microbial, or better still biofilm, growth using larger-scale or realistic environments would be advantageous, the high-throughput, reliable OD-based approach employed here allowed for the testing of a wide range of carbon sources and concentrations to compare the different FPDs, each CAD with its FPD, and the influence of concentration.

Future studies should also aim to expand our understanding of the impacts of FPDs and CADs on a broader spectrum of URBs. This research could include a more diverse selection of taxa and the consideration of polymicrobial communities, which better represent the natural microbial biofilm in these environments. Given the findings from this research on the carbon concentration-dependent growth kinetics of *S. natans*, future work should also examine whether similar trends exist for other bacterial species. Meanwhile, there are many other considerations, such as exploration of the bioavailability and potential toxicity of additives found in CADs; impacts on other aquatic organisms higher up in the food chain; climate change leading to more extreme weather and temperature/rainfall fluctuations; and the role of river flow in URB formation and structuring. Future research should incorporate these climate-related factors to fully understand the evolving impacts on URB growth and other environmental consequences of FPDs.

## 4 Conclusion

In conclusion, this research investigated the influence of various freeze-point depressants (FPDs) and commercial airport de-icers (CADs) on the growth of *S. natans*, as a representative taxon of URBs. The study demonstrated that carbon sources including propylene glycol, ethylene glycol, and sodium acetate fostered *S. natans* growth, highlighting the metabolic versatility of URBs. Furthermore, a negligible difference was observed between pure-FPDs and CADs, indicating that additives in commercial de-icers exert minimal impact on microbial growth.

The research revealed a clear link between carbon concentration and microbial growth kinetics, identifying a limiting concentration, above which the growth rate did not increase significantly. These findings underscore the importance of carbon concentration in controlling microbial growth in environments affected by de-icers. Comparing FPDs sodium acetate had a shorter TTD and faster maxROC than propylene glycol, which was likewise shorter and faster than ethylene glycol. Total growth was highest for propylene glycol. Overall, these findings contribute significantly to our understanding of URBs’ growth response to carbon sources prevalent in airport de-icers. However, further research is needed to expand these findings to other URB taxa, study the impacts of FPDs on mixed species cultures, and explore the potential ecological consequences of these substances in natural aquatic environments. Such insights would further enhance our knowledge of microbial growth kinetics, aiding the development of sustainable management strategies for ecosystems impacted by de-icers.

## Data Availability

The datasets presented in this study can be found in online repositories. The names of the repository/repositories and accession number(s) can be found below: https://doi.org/10.57996/cran.ceres-2630.
