# Supplementary material for: Impact of carbon sources in airport de-icing compounds on the growth of Sphaerotilus natans
Source: Front Microbiol. 2024 Oct 23;15:1320487. doi: 10.3389/fmicb.2024.1320487 (PMC11537983; doi:10.3389/fmicb.2024.1320487)
Supplement: Supplementary file 1 [file Table_1.docx]

**Supplementary Information**

#### Carbon-derived reducing equivalents

Table 6: The carbon-derived reducing equivalents and half reactions for each freeze-point depressant in this study.

| **Freeze-point depressant** | **Carbon derived reducing equivalents** | **Half reactions** |
| --- | --- | --- |
| Propylene glycol | 8e^-^ | C_3_H_8_O_2_ 🡪 3CO_2_ + 8H^+^ + 8e^-^  O_2_ + 4H^+^+ 4e^-^ 🡪 2H_2_O |
| Ethylene glycol | 6e^-^ | 2C_2_H_6_O_2_ 🡪 4CO_2_ + 12H^+^ + 12e^-^  3O_2_ + 12H^+^+ 12e^-^ 🡪 6H_2_O |
| Sodium acetate | 4e^-^ | C_2_H_3_O_2_Na 🡪 2CO_2_ + 3H^+^ + Na^+^ + 4e^-^  O_2_ + 4H^+^+ 4e^-^ 🡪 2H_2_O |
| Potassium formate | 2e^-^ | 2CHO_2_K 🡪 2CO_2_ + 2H^+^ + 2K^+^ + 4e^-^  O_2_ + 4H^+^+ 4e^-^ 🡪 2H_2_O |

#### Comparing the maximum optical density (maxOD, 600nm) relative to different approaches to standardising carbon concentration

There are different valid approaches to standardising carbon concentration outlined in the main text. These include standardising by reducing equivalents, mass/moles of freeze-point depressants (FPDs), or moles of carbon to better compare the microbial growth using different FPDs.

Table 7: The maximum optical density at 600nm (maxOD) per unit of concentration.

|  | **maxOD per reducing equivalent** | **maxOD per mass of FPD (mg/L)** | **maxOD per moles of FPD (mol/L)** | **maxOD per moles of C (mol/L)** |
| --- | --- | --- | --- | --- |
| Propylene glycol | 1.97×10^-3^ | 1.166 | 17.74 | 5.91 |
| Ethylene glycol | 3.49×10^-3^ | 0.845 | 10.49 | 5.25 |
| Sodium acetate | 4.67×10^-3^ | 0.792 | 9.35 | 4.67 |
